# A Pediatric Renal Lymphoma Case Presenting with Central Nervous System Findings

**DOI:** 10.4274/Tjh.03164

**Published:** 2013-06-05

**Authors:** Ahmet Baran, Serhan Küpeli, Ömer Doğru

**Affiliations:** 1 Diyarbakır Children’s Diseases Hospital, Department of Radiology, Diyarbakır, Turkey; 2 Diyarbakır Children’s Diseases Hospital, Department of Pediatric Oncology, Diyarbakır, Turkey; 3 Diyarbakır Children’s Diseases Hospital, Department of Pediatric Hematology, Diyarbakır, Turkey

**Keywords:** Renal lymphoma, Central nervous system involvement, children

## Abstract

In pediatric patients renal lymphoma frequently presents in the form of multiple, bilateral mass lesions, infrequently as a single or retroperitoneal mass, and rarely as diffuse infiltrative lesions. In patients with apparent central nervous system involvement close attention to other physical and laboratory findings are essential for preventing a delay in the final diagnosis. Herein we present a pediatric patient with renal lymphoma that presented with central nervous system findings that caused a delay in diagnosis.

**Conflict of interest:**None declared.

## Abstract (tr)

Çocukluk çağında böbrek kökenli lenfoma sıklıkla bilateral kitle lezyonları, daha az sıklıkla da tek veya retroperitoneal bir kitle olarak veya difüz infiltratif lezyonlar şeklinde dikkate gelebilir. Tanının gecikmemesi adına, santral sinir sistemi tutulumu bulguları olan hastalarda, diğer fizik ve laboratuar bulgularına da yeterince dikkat edilmesi zorunludur. Bu yazıda, renal lenfoması olan ancak santral sinir sistemi bulguları ile prezente olduğundan tanı konulmasında gecikilen bir çocuk hasta sunulmuştur.

## INTRODUCTION

Rapid and accurate diagnosis of non-Hodgkin lowercase L (NHL) the second most common malignant tumor of childhood in developing countries- is important for reducing morbidity and mortality. Radiologically, primary renal lymphoma has a similar appearance in pediatric and adult cases [1,2,3]. Herein we present a pediatric patient with renal lymphoma that presented with central nervous system (CNS) findings, as well as a discussion concerning the delay in diagnosis due to this atypical presentation. 

## CASE

**Figure 1:**
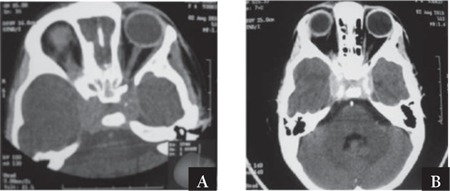
Contrast-enhanced and non-contrast-enhanced axial cranial CT scans. A. Mild hyperdense lesion in the sellar region with well-defined borders. B. Homogeneous significant contrast enhancement and extension of the lesion to the cavernous sinus is seen following intravenous injection of contrast material.

According to the discharge report of a 4-year-old female patient, she presented to a university hospital with a 1-month history of abdominal distention, and a 5-7-d history of headache and inability to move her right eye. Right-eye proptosis and abdominal distention were observed on physical examination. Laboratory findings were as follows: WBC count: 11,400 mm–3 (normal range: 4,000-11,000 mm–3); urea: 65 mg dL–1 (normal range: 10-48 mg dL–1); uric acid: 9.4 mg dL–1 (normal range: 2.2-5.7 mg dL–1); LDH: 1107 U L–1 (normal range: 120-300 U L–1); creatinine: 1.92 mg dL–1 (normal range: 0.6-1.3 mg dL–1). Additionally, cranial CT performed due to ptosis and inability to move the right eye showed a 35x25x15-mm lesion in the sellar region that exhibited homogeneous contrast enhancement with intravenous contrast material (IVCM) (Figure 1). Contrast-enhanced CT angiographic examination showed a hyperdense 37x12x22-mm mass that extended from the sellar region to the parasellar region, surrounding both cavernous sinuses in their entirety, and normal arterial structures in the brain. CT angiography did not show any evidence of thrombus and the patient was referred to our pediatric oncology department with the diagnosis of an intracranial mass. 

**Figure 2:**
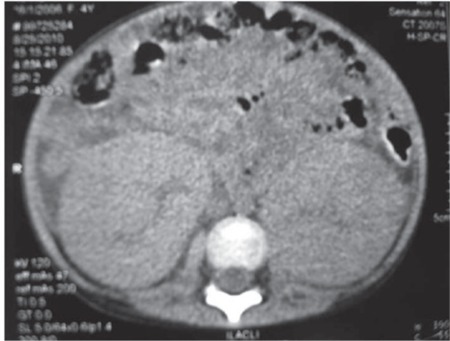
Non-contrast-enhanced axial abdominal CT scan. Significant bilateral kidney enlargement and intraabdominal free fluid are seen.

At the time of admission physical examination of the patient showed pale skin, right-eye proptosis, ptosis, right central facial paralysis, external ophthalmoplegia, abdominal distention, enlarged superficial veins of the abdomen, and an abdominal mass with ill-defined borders. The patient’s uric acid level was 18.9 mg dL-1 and suggestive of tumor lysis syndrome; as such, 1 session of hemodialysis was administered. Thereafter, alkaline fluid, allopurinol treatment, and close electrolyte monitoring were performed, and tumor lysis syndrome prophylactic treatment was given. Abdominal USG examination showed renal enlargement, significant corticomedullary separation, and elevated intraabdominal fluid. Abdominal CT showed bilateral kidney enlargement and free fluid (Figure 2). Peripheral blood smear and bone marrow examination were normal, and Burkitt’s lymphoma was diagnosed following cytological examination of intraabdominal fluid obtained via paracentesis. 

Lumbar puncture showed L3-type blasts in cerebrospinal fluid, which was consistent with Burkitt’s lymphoma. The NHL-BFM 95 chemotherapy protocol was administered to the patient, but following the first course the patient experienced a severe neutropenic sepsis. Meropenem and vancomycin were initially given due to severe febrile neutropenia, and severe oral and anal mucositis. Amphotericin-B and amikacin were added to the therapy on d 3 because of persistent fever, neutropenia, and hypotension, but unfortunately she died on treatment d 5. Pseudomonas aeruginosa was cultured in blood and urine samples obtained during the febrile neutropenic episode. Written informed consent was obtained from the patient’s parents to publish the findings.

## DISCUSSION

Renal lymphoma rarely presents as a single mass lesion in 1 or both kidneys, and may present in the form of a retroperitoneal mass and diffuse renal enlargement [1,2,3]. Following a delay in admission to our pediatric oncology department, the presented patient was observed to have an abdominal mass, which was confirmed radiologically. Radiologically, primary renal lymphoma has a similar appearance in pediatric and adult cases [1,2,3]. Unlike in adults, the pathological diagnosis of renal lymphoma in pediatric cases is commonly Burkitt’s lymphoma. In 6%-70% of pediatric patients it presents in the form of numerous bilateral renal masses, versus a single unilateral lesion or bilateral mass lesions in 10%-20%, and a retroperitoneal mass with diffuse renal enlargement in 

5%-10% [2,3,4]. Diagnosis of Burkitt’s lymphoma in this presented case was based on cytological examination of intraabdominal fluid obtained via paracentesis. Renal biopsy for diagnostic purposes was not performed due to the poor clinical condition of the patient and samples were obtained from intraabdominal and cerebrospinal fluids.

CT findings of renal lymphoma are generally non-specific [1,2,3]. The presented patient’s axial CT scan made without contrast material due to renal insufficiency showed only significant bilateral kidney enlargement and intraabdominal free fluid. Multiple bilateral mass lesions are also observed in leukemia (particularly AML), multiple angiomyolipoma, fungal infections, cystic renal diseases, and metastatic diseases [3]. Benign lesions can be differentiated from malignant renal disease by their particular imaging characteristics: for instance, attenuation in cystic renal disease, presence of fat content in angiomyolipoma, and reduced kidney size in fungal infections. Whereas Wilms’ tumor is observed in young children (0-5 years) with a single renal mass, renal cell carcinoma, metanephric adenoma, solitary angiomyolipoma, and focal infections are observed in older children (after 5 years). Differential diagnosis of a retroperitoneal mass includes retroperitoneal tumors such as neuroblastoma. Diffuse kidney enlargement has numerous causes and its differential diagnosis is difficult. Diffuse renal enlargement can be observed in response to chemotherapeutical agents in patients with tumor lysis syndrome secondary to infections or tumor involvement [5]. 

Palpation of the abdominal mass and an elevated uric acid level were noteworthy in the presented patient, and diagnosis was based paracentesis, despite the absence of enlarged retroperitoneal lymph nodes. Renal insufficiency associated with tumor lysis syndrome is a frequent complication that clinicians must be aware of [6,7,8]. Bilateral primary renal NHL is an extremely rare condition in the pediatric age group and only a few cases have been reported in the English-language medical literature [9,10,11,12]. Primary renal NHL generally presents with an abdominal mass, as in the presented case, but renal failure, hematuria, and anemia have also been described [13,14]. Interestingly, Jindal et al. [12] recently reported a 3-year-old male with bilateral primary renal B-cell lymphoma that presented with orbital metastases, and was characterized by difficulty in diagnosis and management similar to that in the presented patient. The overall prognosis in patients with bilateral primary renal NHL is poor, especially in those that present with renal failure.

In pediatric lymphoma cases the presence of renal disease at the time of diagnosis does not indicate a poor prognosis, but recurrence is associated with a 5-year survival rate of only 30% [15]. Primary renal lymphoma-especially in association with central nervous system involvement-is a rare condition. In the presented case the focus of attention on the central nervous system delayed the diagnosis. In particular, in cases with concomitant elevated uric acid and nephromegaly the diagnosis of renal lymphoma should always be a consideration. 

## CONFLICT OF INTEREST STATEMENT

The authors of this paper have no conflicts of interest, including specific financial interests, relationships, and/ or affiliations relevant to the subject matter or materials included.
